# Chitosan@Carboxymethylcellulose/CuO-Co_2_O_3_ Nanoadsorbent as a Super Catalyst for the Removal of Water Pollutants

**DOI:** 10.3390/gels8020091

**Published:** 2022-02-03

**Authors:** Nujud Maslamani, Esraa M. Bakhsh, Sher Bahadar Khan, Ekram Y. Danish, Kalsoom Akhtar, Taghreed M. Fagieh, Xintai Su, Abdullah M. Asiri

**Affiliations:** 1 Chemistry Department, Faculty of Science, King Abdulaziz University, Jeddah 21589, Saudi Arabia; nujudomar86@gmail.com (N.M.); eydanish@kau.edu.sa (E.Y.D.); kaskhan@kau.edu.sa (K.A.); tfagieh@kau.edu.sa (T.M.F.); aasiri2@kau.edu.sa (A.M.A.); 2 Center of Excellence for Advanced Materials Research, King Abdulaziz University, Jeddah 21589, Saudi Arabia; 3 Guangdong Provincial Key Laboratory of Solid Wastes Pollution Control and Recycling, School of Environment and Energy, South China University of Technology, Guangzhou 510006, China; suxintai@scut.edu.cn

**Keywords:** chitosan, CMC, CuO-Co_2_O_3_, nanocomposite beads, inorganic and organic pollutants

## Abstract

In this work, an efficient nanocatalyst was developed based on nanoadsorbent beads. Herein, carboxymethyl cellulose–copper oxide-cobalt oxide nanocomposite beads (CMC/CuO-Co_2_O_3_) crosslinked by using AlCl_3_ were successfully prepared. The beads were then coated with chitosan (Cs), Cs@CMC/CuO-Co_2_O_3._ The prepared beads, CMC/CuO-Co_2_O_3_ and Cs@CMC/CuO-Co_2_O_3_, were utilized as adsorbents for heavy metal ions (Ni, Fe, Ag and Zn). By using CMC/CuO-Co_2_O_3_ and Cs@CMC/CuO-Co_2_O_3_, the distribution coefficients (K_d_) for Ni, Fe, Ag and Zn were (41.166 and 6173.6 mLg^−1^), (136.3 and 1500 mLg^−1^), (20,739.1 and 1941.1 mLg^−1^) and (86.9 and 2333.3 mLg^−1^), respectively. Thus, Ni was highly adsorbed by Cs@CMC/CuO-Co_2_O_3_ beads. The metal ion adsorbed on the beads were converted into nanoparticles by treating with reducing agent (NaBH_4_) and named Ni/Cs@CMC/CuO-Co_2_O_3_. Further, the prepared nanoparticles-decorated beads (Ni/Cs@CMC/CuO-Co_2_O_3_) were utilized as nanocatalysts for the reduction of organic and inorganic pollutants (4-nitophenol, MO, EY dyes and potassium ferricyanide K_3_[Fe(CN)_6_]) in the presence of NaBH_4_. Among all catalysts, Ni/Cs@CMC/CuO-Co_2_O_3_ had the highest catalytic activity toward MO, EY and K_3_[Fe(CN)_6_], removing up to 98% in 2.0 min, 90 % in 6.0 min and 91% in 6.0 min, respectively. The reduction rate constants of MO, EY, 4-NP and K_3_[Fe(CN)_6_] were 1.06 × 10^−1^, 4.58 × 10^−3^, 4.26 × 10^−3^ and 5.1 × 10^−3^ s^−1^, respectively. Additionally, the catalytic activity of the Ni/Cs@CMC/CuO-Co_2_O_3_ beads was effectively optimized. The stability and recyclability of the beads were tested up to five times for the catalytic reduction of MO, EY and K_3_[Fe(CN)_6_]. It was confirmed that the designed nanocomposite beads are ecofriendly and efficient with high strength and stability as catalysts for the reduction of organic and inorganic pollutants.

## 1. Introduction

Water pollution and its treatment is one of the most serious issues worldwide. Effluent discharge is produced from human activities including industrialization, which introduces a considerable amount of wastewater into nature [1]. Organic contaminates are the most noticeable water pollutants (e.g., organic dyes and nitrophenol compounds), which have deteriorating effects on human health and other living organisms due to their carcinogenic effect on nature [2]. Beside organic contaminations, inorganic pollutants are also considered as the most serious environmental problem including heavy metal ions. According to the literature, the accumulation of toxic metal ions in wastewater can cause genetic alteration, which can affect hormone metabolism and lead to serious diseases such as cancer and fetal growth restriction. As reported in the literature, potassium hexacyanoferrate (III) is another example of an inorganic pollutant; it can cause acute toxicity and carcinogenicity even at trace levels [3,4].

Various procedures have been reported as effective methods for pollutants’ removal from wastewater such as reverse osmosis, distillation, ion-exchange, coagulation–flocculation, filtration, extraction and adsorption by nanoscale materials [5,6,7]. Recently, newly designed cost-effective, economic, nontoxic and productive nanoscale materials have been gained attention in various scientific areas such as the water treatment, drug delivery and solar energy conversion fields. Adsorbent-based nanoscale materials are a highly effective way to remove contaminates from wastewater [8,9,10]. Due to their ease of operation, convenience, low-cost and high efficiency with respect to the removal of metal ions, minerals [11], activated carbon [12], agricultural waste [13], metal oxide nanoparticles, etc., are widely utilized as adsorbents. Among various nanoadsorbents, metal oxide is potentially an excellent nanoadsorbent due to its unique optical properties, large surface area and low toxicity, thus showing great potential in water treatment and high activities [14]. However, combinations of metal oxide nanoparticles come across two main obstacles in their application. Metal oxide nanoparticles tend to react with the surrounding media, and aggregation easily occurs due of van der Waals interactions or agglomerates, leading to a loss in their activity [15,16]. Separation of metal oxide nanoparticles from solutions for recycling is another obstacle due to their small size. To solve this problem, metal oxide nanoparticles can be coated with a supported polymer.

Eco-friendly polymeric materials have been rapidly developed for broad range of applications and attracted much attention due to their unique proprieties, including improvement of the efficiency of adsorption, cost effectiveness, biocompatibility and their promising results in the treatment of wastewater [17,18,19]. Among many other polymers, carboxymethylcellulose (CMC) is a biopolymer of a cellulose derivative with multiple carboxylic and hydroxyl functional groups. CMC has been applied in removal of dyes and metal ions due to its low cost, nontoxicity, biodegradability, biocompatibility and renewability [4,20].There is one limitation of pure CMC, which is its low mechanical strength. However, this drawback could be overcome by an addition of nanofillers or nanocomposites to the CMC polymer. In recent years, researchers have focused on a combination of CMC-nanocomposite beads due to their superior properties. CMC-nanocomposite beads are physically or chemically crosslinked with trivalent metals such as Fe^+3^ or Al^+3^, forming coordination bonds between metal ions and (–COOH) in the polymer side chain [4,21,22].

Chitosan with aminopolysaccharide is another biodegradable polymer, which has been vastly studied for various applications due to its great characteristics (e.g., nontoxicity, cost effectiveness, high water permeability, adhesion, cross-linker agent and good coating ability). It has been reported that it has high adsorption aptitude as an adsorbent of heavy metal ions because it has specific physicochemical characteristics (e.g., high hydrophilicity, excellent chelation performance, great number of adsorption sites, high reactivity and selectivity toward metal ions) [23,24]. Moreover, the NH_2_ and OH groups are the main reactive groups, which are responsible for adsorption of metal ions. Thus, chitosan coating has played a significant role in stabilizing the nanoparticles by coating the surface of the nanosized metal oxide nanoparticles to prevent agglomeration during the adsorption process [25].

Many efforts have been carried out toward the development of CuO and Co_2_O_3_ as a nanoadsorbent and a nanocatalyst [16,26]. At the same time, extensive efforts have been conducted toward the development of different nanocomposites for different applications [27,28,29,30,31,32,33,34]. Based on our survey, CuO-Co_2_O_3_ with a combination of CMC and a chitosan coating has not been reported yet. Thus, the combination of novel, facile and green adsorbent nanocomposite beads (CMC/CuO-Co_2_O_3_) with a chitosan coating to form Cs@CMC/CuO-Co_2_O_3_ beads was successfully prepared. Both adsorbents were utilized for the removal of metal ions (Ni, Zn, Ag and Fe). In order to recycle the beads, Ni(II)/Cs@CMC/CuO-Co_2_O_3_ and Ag(I)/CMC/CuO-Co_2_O_3_ were studied. They were treated by NaBH_4_ for the complete conversion to form Ni(0)/Cs@CMC/CuO-Co_2_O_3_ and Ag(0)/CMC/CuO-Co_2_O_3_ nanoparticles and then utilized as efficient catalysts for reduction of 4-NP, MO, EY and K_3_[Fe(CN)_6_]. The prepared nanocomposite beads and the beads were characterized by various analytical techniques, including FT-IR, SEM, EDX and XRD.

## 2. Result and Discussion

### 2.1. Characterization

#### 2.1.1. Field-Emission Scanning Electron Microscope (FE-SEM) Analysis

**Figure 1:**
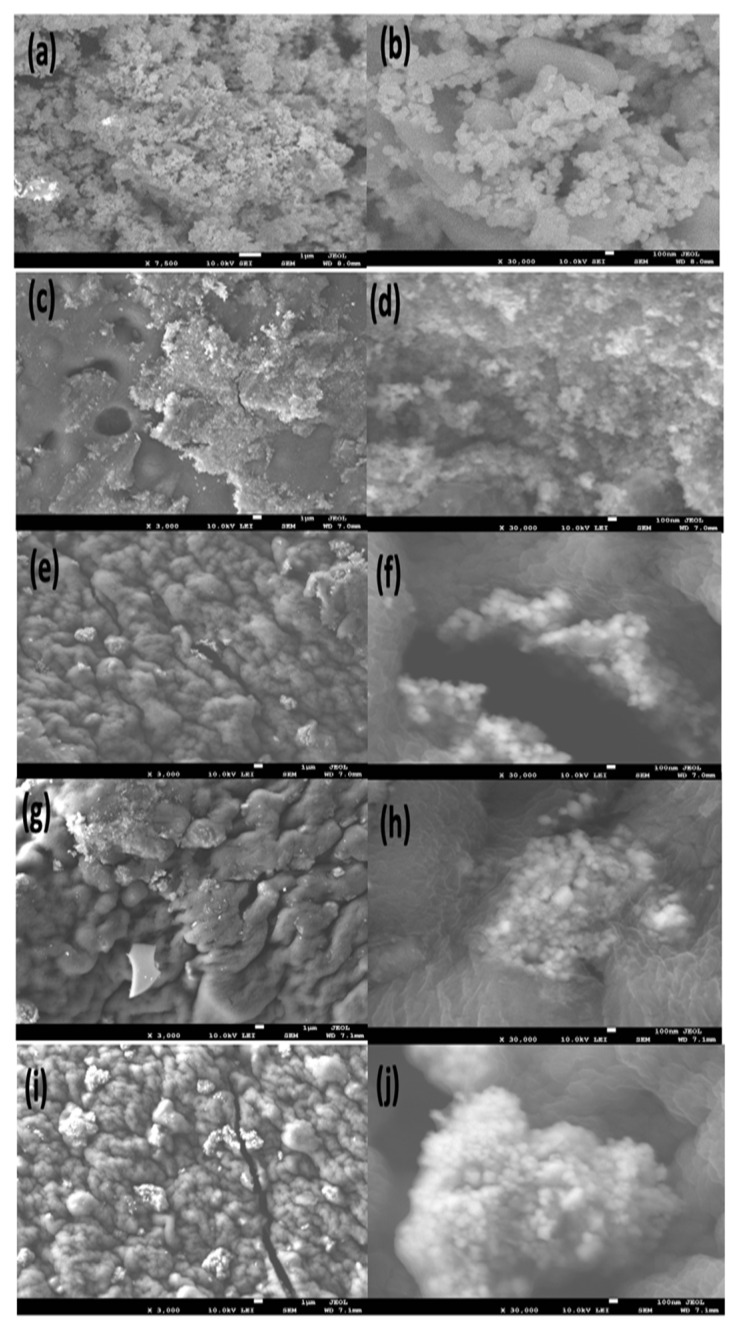
FE-SEM images of (**a**,**b**) CuO-Co_2_O_3_, (**c,d**) CMC, (**e,f**) CMC/CuO-Co_2_O_3_, (**g,h**) Cs@ CMC/CuO-Co_2_O_3_ and (**i,j**) Ni/Cs@ CMC/CuO-Co_2_O_3_.

The surface morphology of the prepared materials, CuO-Co_2_O_3_, CMC, CMC/CuO-Co_2_O_3_, Cs@CMC/CuO-Co_2_O_3_ and Ni/Cs@CMC/CuO-Co_2_O_3_, was examined by utilizing FE-SEM, as seen in Figure 1. The low-magnification and high-magnification images for the prepared nanocomposite beads are represented on the left and right sides in Figure 1. The CuO-Co_2_O_3_ picture indicates the particles of CuO-Co_2_O_3_, in Figure 1a,b. The pure CMC beads showed flat surfaces with less porosity [4,35], as seen in Figure 1c,d. On the other hand, CMC/CuO-Co_2_O_3_ images illustrated that CuO-Co_2_O_3_ was planted very well on the CMC surface, as seen in Figure 1e,f. As seen in Figure 1g,h for Cs@CMC/CuO-Co_2_O_3_, chitosan was coated and filled the porous surface of the CMC/CuO-Co_2_O_3_. Moreover, Figure 1i,j illustrates that Ni was rooted and dispersed on Cs@CMC/CuO-Co_2_O_3_, covering most of the Cs@CMC/CuO-Co_2_O_3_ surface. The surface area of the metal increased due to the presence of some functional groups such as COO– on CMC, and N-H_2_ and O–H on chitosan [36].

#### 2.1.2. Energy Dispersive X-ray (EDX) Analysis

**Figure 2:**
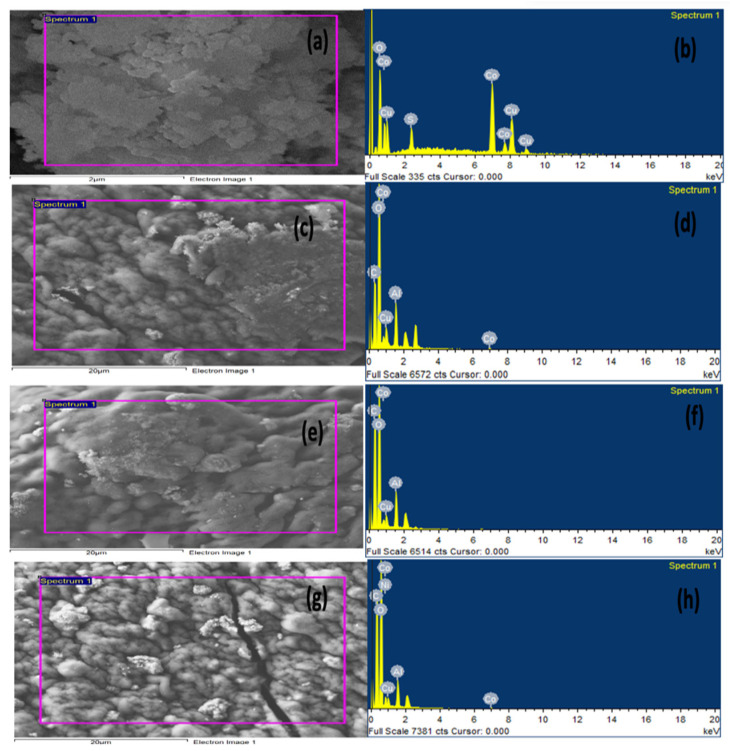
EDX spectra of (**a**,**b**) CuO-Co_2_O_3_, (**c,d**) CMC/CuO-Co_2_O_3_, (**e,f**) Cs@CMC/CuO-Co_2_O_3_ and (**g,h**) Ni/Cs@CMC/CuO-Co_2_O_3_.

For more confirmation of the prepared nanocomposite beads, EDX analysis was applied. As clearly seen from Figure 2, the Cu and Co elements were present in the powder CuO-Co_2_O_3_ and in all prepared beads (CMC/CuO-Co_2_O_3_, Cs@CMC/CuO-Co_2_O_3_ and Ni/Cs@CMC/CuO-Co_2_O_3_), confirming the successful synthesis of proposed materials. The carbon and oxygen elements were present in EDX images for CMC/CuO-Co_2_O_3_, Cs@CMC/CuO-Co_2_O_3_ and Ni/Cs@CMC/CuO-Co_2_O_3_ due to chitosan and CMC functional groups [37]. Al element was observed in all beads because AlCl_3_ was used as a cross-linking agent in the formation of the beads.

#### 2.1.3. X-ray Diffraction (XRD) Analysis

**Figure 3:**
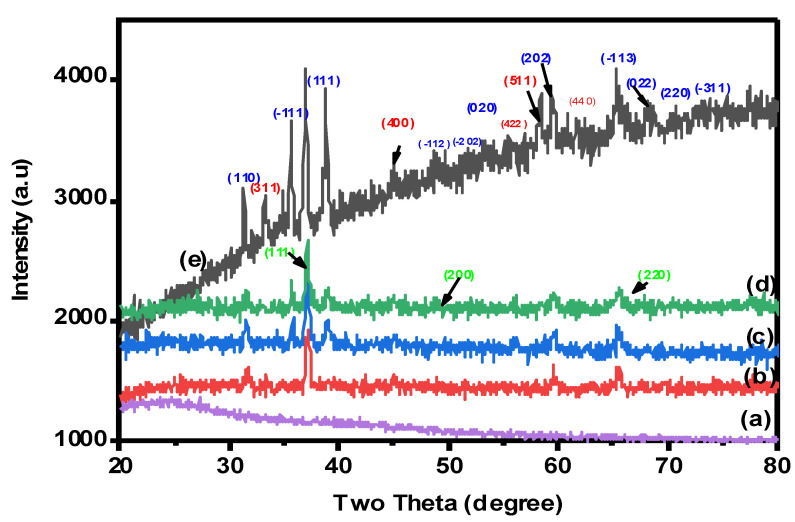
XRD patterns for (**a**) CMC, (**b**) CMC/CuO-Co_2_O_3_, (**c**) Cs@CMC/CuO-Co_2_O_3_, (**d**) Ni/Cs@CMC/CuO-Co_2_O_3_ and (**e**) CuO-Co_2_O_3_.

The crystal structures and phase purity of nanocomposite beads were examined by XRD analysis. The X-ray diffraction patterns were collected in the 2θ range of 10–80°, as clearly indicated in Figure 3. As seen in Figure 3a, the XRD pattern of pure CMC showed a broad peak located at 2θ = 22 owing to the amorphous CMC crystal [4]. The spectrum of XRD for the CuO-Co_2_O_3_ nanocomposite (Figure 3e) showed diffraction bands at 2θ = 35.56, 38.64, 48.84, 49.2, 60.4, 65.0, 67.5, 70.3 and 75.2, confirming the monoclinic structure of CuO, which were found at (110), (−111), (111), (−112), (−202), (020), (202) and (−311) [4]. The diffraction peaks at (311), (400), (511), (220) and (440) are responsible for the Co_2_O_3_ phase [38]. Therefore, CMC/CuO-Co_2_O_3_, Cs@CMC/CuO-Co_2_O_3_ and Ni/CMC/CuO-Co_2_O_3_ have the same diffraction peaks of the CuO-Co_2_O_3_ nanocomposite, indicating the successful preparation of the beads, as clearly seen in Figure 3b–d. Moreover, the XRD patterns of Ni/Cs@CMC/CuO-Co_2_O_3_ beads showed peaks located at (111), (200) and (220), which are attributed to the Ni phase on the surface of Ni/Cs@CMC/CuO-Co_2_O_3_ beads.

#### 2.1.4. FT-IR Analysis

**Figure 4:**
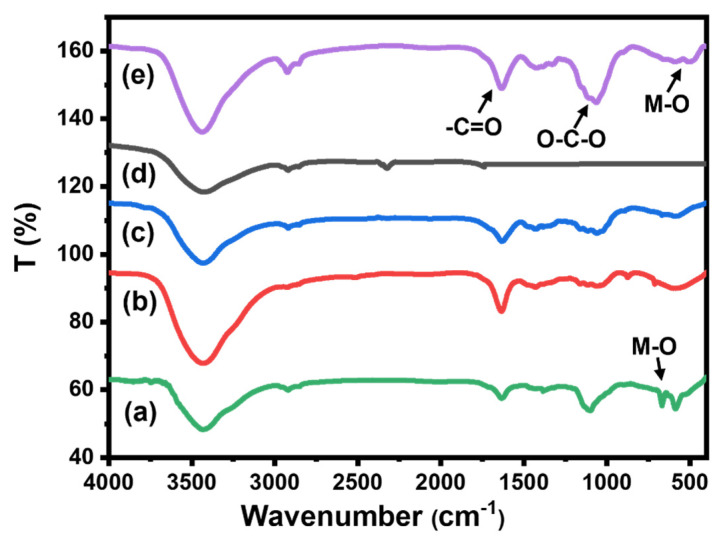
FT-IR spectra for (**a**) CuO-Co_2_O_3_, (**b**) pure CMC, (**c**) CMC/CuO-Co_2_O_3_, (**d**) Cs@CMC/CuO-Co_2_O_3_ and (**e**) Ni/Cs@CMC/CuO-Co_2_O_3_.

Figure 4 represents the FT-IR spectra of all prepared beads, pure CMC, CMC/CuO-Co_2_O_3_, Cs@CMC/CuO-Co_2_O_3_ and Ni/Cs@CMC/CuO-Co_2_O_3_ along with CMC/CuO-Co_2_O_3_ powder. The spectrum of CuO-Co_2_O_3_ (Figure 4a) illustrated a band at 400–600 cm^−1^, which was assigned to the metal oxides (M–O); besides a broad band for O–H (bending and stretching) [4]. FT-IR spectrum of CMC (Figure 4b) exhibited broad bands at 3300–3500 cm^−1^, 1422 and 1607, and 1000 and 1200 cm^−1^, which were assigned to the stretching of -OH groups, symmetrical and asymmetrical stretching vibrations of the COO– groups and –C–O stretching on the polysaccharide skeleton, respectively [4,39]. All the bands were present in CMC/CuO-Co_2_O_3_ (Figure 4c), while for chitosan coating beads Cs@CMC/CuO-Co_2_O_3_ (Figure 4d) new bands at 1155 and 1654 cm^−1^ appeared, which were attributed to the saccharide and (–NH_2_) amine group in chitosan polymer [40]. All the peaks that appeared in the Ni/Cs@ CMC/CuO-Co_2_O_3_ beads are clearly seen in Figure 4e.

### 2.2. Metal Uptake Study

To evaluate the amount of metal adsorbed on the surface of Cs@CMC/CuO-Co_2_O_3_ and CMC/CuO-Co_2_O_3_ beads, distribution coefficient (*K_d_*) and uptake capacity (q_e_) were calculated using the following Equations (1) and (2) [41]:

(1) $$K_{d}=\frac{\left(Ci-Ce\right)}{Ce}*\frac{V\left(ml\right)}{m\left(g\right)}$$

(2) $$q_{e}=\frac{\left(Ci-Ce\right)*V\left(L\right)}{m\left(g\right)}$$

where *C_i_* and *C_e_* are the concentration of the metal ions before and after adsorption by Cs@CMC/CuO-Co_2_O_3_ and CMC/CuO-Co_2_O_3_ nanocomposite beads, respectively. *V* refers to the solution volume (L), and m is the mass of beads (g).

**Figure 5:**
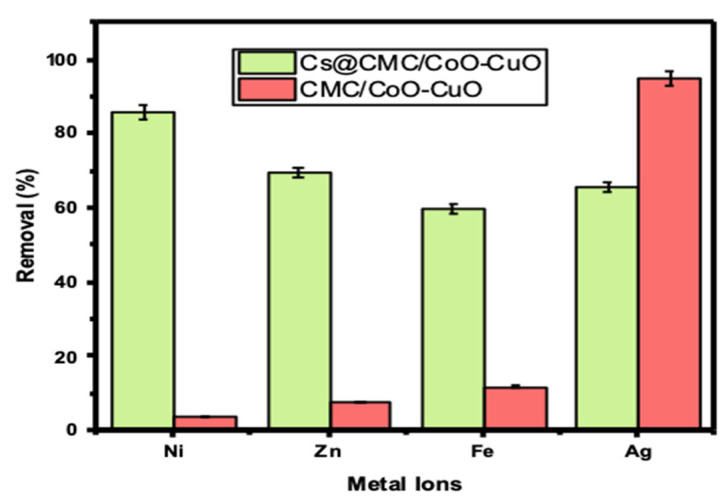
Removal percentage of metal ions using 5 mg of CMC/CuO-Co_2_O_3_ and Cs@CMC/CuO-Co_2_O_3_ vigorously shaken for 60 min at RT (25 ± 1 °C).

As can be seen from Table 1, Ni has the highest removal percentage with Cs@CMC/CuO-Co_2_O_3_ compared to other metals, (66, 86.06, 70 and 79%) for Ag(I), Ni(II), Zn(II) and Fe(II), respectively. When compared the adsorbed metals, Ag(I) has the highest adsorption (%) with CMC/CuO-Co_2_O_3_ up to 95%. This result indicates that Ag(I) was more adsorbed by CMC/CuO-Co_2_O_3_ beads, and Ni(II) was more adsorbed by Cs@CMC/CuO-Co_2_O_3_ beads. However, the Cs@CMC/CuO-Co_2_O_3_ adsorbent was more effective than the CMC/CuO-Co_2_O_3_ beads toward all metals, as shown in Figure 5. The reason for this is that chitosan has strong chelating proprieties toward metal ions due to the high content of amino and hydroxyl groups present in the composition of chitosan, which act as active sites [15,42,43].

### 2.3. Optimization of Ni(II) Adsorption

#### 2.3.1. Effect of Initial Concentration of Ni(II) Solution

**Figure 6:**
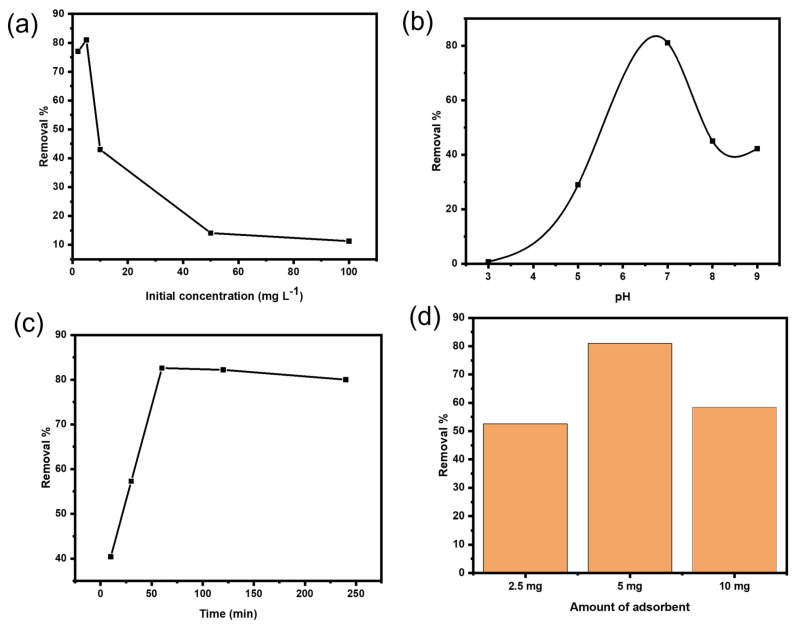
(**a**) Effect of Ni(II) initial concentration using 5 mg Cs@CMC/CuO-Co_2_O_3_ at pH = 7. (**b**) Effect of pH using 5 mg L^−1^ Ni(II) and 5 mg Cs@CMC/CuO-Co_2_O_3._ (**c**) Effect of contact time using 5 mg L^−1^ Ni(II) and 5 mg Cs@CMC/CuO-Co_2_O_3_ at pH = 7. (**d**) Effect of Cs@CMC/CuO-Co_2_O_3_ dose at pH = 7.

As shown in Figure 6a, the influence of different concentrations of Ni(II) ions was tested to evaluate the adsorption isotherms. According to literature, the adsorption efficiency decreases with the increase in Ni(II) concentration as proved in most studies on heavy metals removal. The possible explanation is that the adsorbent surface sites are enough to accommodate the metal ions in solution and the sorption rate gets faster. However, when the metal concentrations are increased, the adsorbent surface sites will not be enough to capture all the metal ions present in the solution [44].

#### 2.3.2. Effect of pH of Ni(II) Solution

The effect of pH was the most significant controlling parameter in the adsorption study. Adsorption of heavy metals depends on the pH and the type of ions solution. For heavy metal adsorption, a pH range of 5.0–8.0 is usually sufficient. Due to the decrease in H^+^ concentration, heavy metal ions exist as free ions with an initial pH range of 4.0–5.0 and can be adsorbed onto chitosan at higher pH values. Because H^+^ concentration is high at lower pH values, protonation of amino groups can cause electrostatic repulsion between protonated group and heavy metal ions. The net negative charge on the surface of chitosan increases when the pH value rises, and the ionic point of ligands such as –COOH, –OH and –NH_2_ groups becomes free, enhancing binding with the heavy metal ions. It has been reported that at pH values less than 6.5, chitosan has strong cationic charges and strong anionic charges at pH higher than 6.5. In our adsorbent beads, the carboxylic (–COOH) and amino (–NH_2_) groups existing in the beads are responsible for the binding of Ni(II). Therefore, various values of pH for Ni ions solution were examined using the adsorbent Cs@CMC/CuO-Co_2_O_3_ nanocomposite beads, as illustrated in Figure 6b. It was clearly found that the adsorption of Ni ions increased with an increase in pH from 3 to 7 (neutral); and the removal efficiency was 1%, 29% and 83% for pH values 3, 5 and 7, respectively. However, the adsorption then reduced greatly with an increase in the pH from 7 to 9. The reduction in adsorption at high pH may be either due to aggregation of chitosan polymer because of the hard protonation of its amino groups, or may be due to the precipitation of metal ions or Ni ions in the alkaline medium as Ni(OH)_2_. [45]. Therefore, the neutral pH was selected as an optimal condition for further experiments. The same effect was reported in the literature [26,46,47].

#### 2.3.3. Effect of Ni(II) Adsorption Contact Time

The effect of contact time is a significant factor in adsorption study. In fact, the adsorption property is very dependent on the time required for equilibrium between the adsorbent and adsorbate. The adsorption of Ni ions was carried out by applying different contact times (10, 30, 60, 120, 240 min). As clearly seen from Figure 6c, 48.6% of Ni(II) was removed in 10 min and reached 88.7% in 60 min before it gradually decreased to 62% at 240 min. The explanation for this phenomenon is that in the first 60 min, the surface of the Cs@CMC/CuO-Co_2_O_3_ was filled with the Ni(II), and the whole surface was occupied, but the adsorption process was gradually reduced after 60 min due to the saturation of active sites on the beads’ surface. This result is in accordance with previous published findings [48]. 

#### 2.3.4. Effect of Adsorbent Dose

The adsorbent dose is also an important factor in adsorption. To evaluate the effect of the adsorbent amount on Ni removal, three different doses of CS@CMC/CuO-Co_2_O_3_ beads (2.5, 5 and 10 mg) were used for a fixed initial Ni(II) concentration (5 mg L^−1^) at 25 °C with a contact time of 60 min. As clearly seen in Figure 6d, the removal percentage of Ni(II) was increased from 53% to 83% when the number of beads increased from 2.5 to 5 mg, respectively. However, the removal percentage was then decreased with an increase in bead dosage from 5 to 10 mg. The best explanation for this phenomenon is that the CS@CMC/CuO-Co_2_O_3_ beads have more active sites, which remined unsaturated during the adsorption procedure. Therefore, 5 mg was fixed for further study as the optimum adsorbent dose.

Moreover, the isotherm and kinetic adsorption were studied as follows: The two isotherm models, Langmuir and Freundlich, are given in Table 2 (Equations (4) and (5)) to model the adsorption process of our system. According to the data obtained, the correlation coefficient (R^2^) values for isotherm models, Langmuir was discovered to be a suitable model to represent the sorption system, which assumed a monolayer of analyte establisher on a homogeneous surface of the adsorbent. Linearity of plotting C_e_/q_e_ vs. C_e_ was achieved with R^2^ of 0.9433. The Langmuir constant ($q_{m})$ was calculated to be 12.00 mg g^−1^, which is close to the experimental value of the adsorption capacity (11.00 mg g^−1^). The Langmuir constant (b) is equal to 0.08 L mg^−1^, which explains the strong affinity of the Ni(II) ions to the adsorbent beads. The essential factor R_L_ was calculated using Equation (3) [44].

(3) $$R_{L}=\frac{1}{1+bC_{o}}$$

where $C_{o}$ is the initial concentration of Ni(II) (mgL^−1^), and b is the Langmuir constant. The calculated R_L_ was found to be 0.50, which is in the range of 0 < R_L_ > 1, referring to favorable adsorption. Thus, this confirmed that the adsorption of Ni(II) ions by Cs@CMC/CuO-Co_2_O_3_ is favorable, as shown in Table 3. 

The pseudo-first-order model and second-order model were applied to the adsorption system for an explanation of kinetics, as shown in Table 2 (Equations (6) and (7)). Both slope and intercept were calculated using the plot log ($q_{e}$ − $q_{t})$ vs. t, and t/qt vs. t for the pseudo-first order and the pseudo-second order, respectively. Based on the calculated values, the pseudo-first-order more suitably explained the adsorption. The pseudo-second-order model was close to the adsorption capacities obtained from experiments; Langmuir isotherm and pseudo-second-order kinetic models were compatible, as illustrated in Table 4. The obtained data were compared with other studies for removal of Ni(II), as represented in Table 5.

### 2.4. Adsorption Mechanism

**Figure 7:**
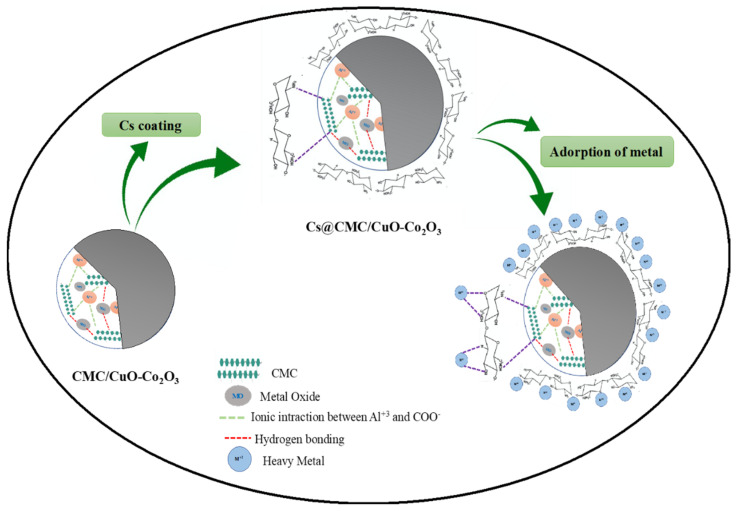
Adsorption mechanism of Cs@CMC/CuO-Co_2_O_3_ nanocomposite beads with metal ions.

The possible adsorption mechanism is illustrated in Figure 7. The adsorption of heavy metals with Cs@CMC/CuO-Co_2_O_3_ might be due to strong attraction of metal ions to nanocomposite beads, which contain active sites (COO–, OH, Cu–O, Co–O, –O– and –NH_2_). These groups can easily attract and combined with metal ions. However, the amino group in chitosan has a significant role in the adsorption because the chitosan completely coats the surface of CMC/CuO-Co_2_O_3_. The chemical nature of chitosan, hydrophilicity due to the large number of (–OH) and the presence of (–NH_2_) can determine the adsorption of chitosan toward the heavy metal. According to the literature, the adsorption of heavy metal ions by chitosan functional groups can occur based on different mechanisms (e.g., electrostatic attraction and chelation). Chitosan (NH_2_) groups are responsible for the adsorption of metal cations by a chelation mechanism. In fact, the adsorption can be affected by the pH of the metal ion solution where the NH_2_ group (free-electron doublet on nitrogen) can attract cations at neutral pH [24,54,55].

### 2.5. Catalytic Reduction Study

**Figure 8:**
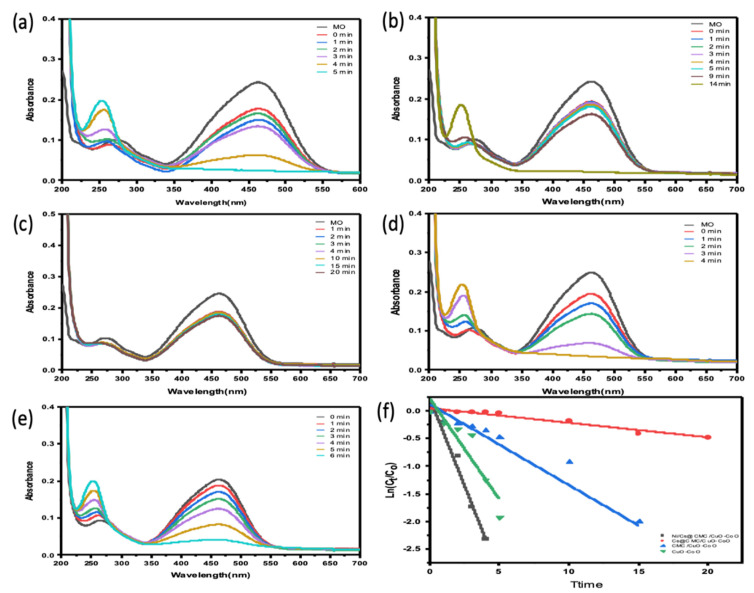
Catalytic activity for the reduction of MO degradation with 5 mg of different catalysts: (**a**) CuO-Co_2_O_3_, (**b**) CMC/CuO-Co_2_O_3_, (**c**) Cs@CMC/CuO-Co_2_O_3_, (**d**) Ni/Cs@CMC/CuO-Co_2_O_3_ and (**e**) Ag/CMC/CuO-Co_2_O_3_. (**f**) ln(Ct/Co) versus time for the transformation of MO using prepared catalysts.

The catalytic ability of all prepared catalysts, including CuO-Co_2_O_3_, CMC/CuO-Co_2_O_3_, Cs@CMC/CuO-Co_2_O_3_ and Ni/Cs@CMC/CuO-Co_2_O_3_, was examined for the reduction of two anionic dyes (MO and EY). MO dye was chosen as a model dye for this study. MO solution (0.01 mM) was placed into a UV-cuvette and mixed with a reducing agent, NaBH_4_. Further, each catalyst was added to the mixture of MO and NaBH_4_ as mentioned previously. Afterward, the reduction reaction of MO was examined by the UV–Vis spectrophotometer every min as the reaction proceeded. Initially, pure MO solution (0.01 mM) was recorded by UV–Vis, and two absorbance bands appeared at λ_max_ = 460 nm and 270 nm, as shown in Figure 8. There was no change observed when the reducing agent (NaBH_4_) was added because NaBH_4_ cannot reduce the dye even with an excess amount, as reported in the literature [56]. However, after the addition of both reducing agent and catalysts, the color of MO dye disappeared gradually from orange to colorless. The reason for this that MO was converted to hydrazine derivatives by breaking the azo bond (-N=N-) and transforming it to -NH_2_ (amino). During the reduction, the peak at l_max_ = 460 nm was decreased gradually [57,58]. The reduction percentage of MO was 92 % in 4 min with Ni/Cs@CMC/CuO-Co_2_O_3_ while it reached to 85%, 90% and 33.5 % with CuO-Co_2_O_3_, CMC/CuO-Co_2_O_3_ and Cs@CMC/CuO-Co_2_O_3_ in 5, 20 and 14 min, respectively. Moreover, the reduction of MO was studied by using the Ag/CMC/CuO-Co_2_O_3_ under the same conditions. It was found that Ag/Cs@CMC/CuO-Co_2_O_3_ can reduce 88% of MO in 6 min.

The kinetic behavior of four prepared catalysts toward the reduction of MO dye was evaluated by applying the pseudo-first-order kinetics. The rate constants were calculated from the slope of lnC_t/_C_0_ vs. time, as seen in Figure 8f. The rate constant *K* value per second and R^2^ correlation coefficient of decolorization of MO dye with Ni/Cs@CMC/CuO-Co_2_O_3_ was 1.02 × 10^−2^ s^−1^ and 0.964, respectively, which is higher than other catalysts, using Cs@CMC/CuO-Co_2_O_3_ (4.4 × 10^−4^ and 0.953), CMC/CuO-Co_2_O_3_ (2.6 × 10^−3^ and 0.915) and CuO-Co_2_O_3_ (6.11 × 10^−3^ and 0.868). This clearly indicates that Ni/Cs@CMC/CuO-Co_2_O_3_ is the most active catalyst among other prepared catalysts toward the reduction of MO dye.

**Figure 9:**
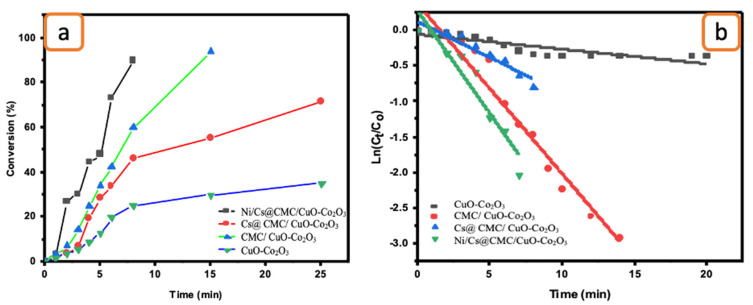
(**a**) Conversion versus time for the reduction of EY by the prepared catalysts. (**b**) ln(C_t_/C_o_) vs. time for the transformation of EY by the prepared catalysts.

Moreover, the catalytic reduction was tested toward the degradation of EY. The decolorization of EY was conducted by using the same procedure described previously for catalytic reduction of MO (Figure 9). EY had an absorbance band at 510 nm, which gradually decreased. During the reduction, the EY color changed from orange to pale yellow and then turned to colorless, indicating the formation of ESH_2_ [59]. Ni/Cs@ CMC/CuO-Co_2_O_3_ was the highest efficient catalyst toward EY. According to data obtained, around 90% of EY was decolorized in 9 min by Ni/Cs@CMC/CuO-Co_2_O_3_, while reduction of 71%, 94% and 35% were obtained in 24, 15 and 25 min with Cs@CMC/CuO-Co_2_O_3_, CMC/CuO-Co_2_O_3_ and CuO-Co_2_O_3_, respectively. The rate constant and R^2^ were found to be 4.58 × 10^−3^ and 0.936, 7.71 × 10^−4^ and 0.968, 3.2 × 10^−3^ and 0.928 and 2.95 × 10^−4^ and 0.989 for Ni/Cs@CMC/CuO-Co_2_O_3_, Cs@CMC/CuO-Co_2_O_3_, CMC/CuO-Co_2_O_3_ and CuO-Co_2_O_3_, respectively (Table 6). Among all prepared catalysts, Ni/Cs@CMC/CuO-Co_2_O_3_ is the most active catalyst toward EY dye. The catalytic activity of the Ni/Cs@CMC/CuO-Co_2_O_3_ toward MO and EY was compared with other reported catalysts in the literature, as illustrated in Table 7.

**Figure 10:**
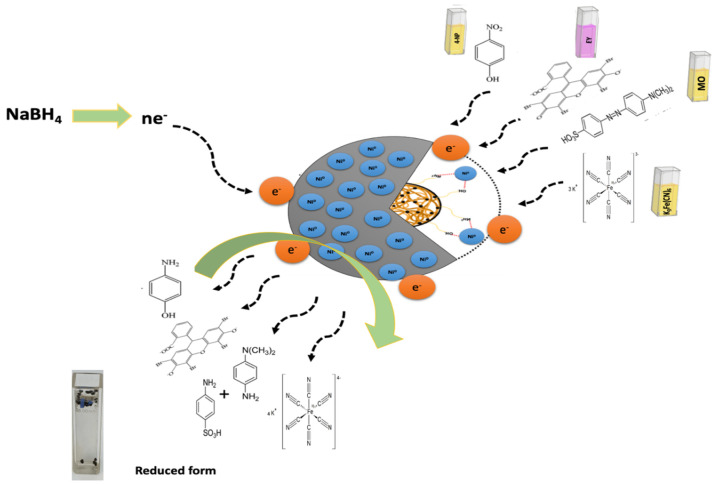
Mechanism for catalytic reduction of target pollutants (4-NP, K_3_[Fe(CN)_6_], MO and EY).

Figure 10 shows the possible reduction mechanism of MO and EY. The reduction occurs mainly through the transfer of electrons via nanocatalyst facilitation. Firstly, the NaBH_4_ dissociates to BH_4_^−^ ions and Na^+^, in which BH_4_^−^ acts as a source of e^−^ and H^+^. Further, the catalyst Ni/Cs@CMC/CuO-Co_2_O_3_ transfers e^−^ from the BH_4_^−^ ion to dye molecules for catalytic reduction. For MO dye, the azo bonds are activated by the electron transfers by BH_4_^−^ ion via Ni/Cs@CMC/CuO-Co_2_O_3_ nanocomposite beads. The MO molecules are bounded to the nanocomposite beads by oxygen and sulfur atoms. Thus, the first step is the conversion of the –N=N– bond into –HN–NH– bond followed by bond-breaking to form aromatic amines. In fact, this happens because e^−^ are accepted from nanocomposite beads catalyst and H^+^ from BH_4_^−^. Thus, the orange color of the MO dye is turned colorless, indicating the completion of the reduction of MO. On the other hand, the EY is adsorbed on the surface of Ni/Cs@CMC/CuO-Co_2_O_3_ nanocomposite beads because of the electrostatic attractive force between Ni/Cs@CMC/CuO-Co_2_O_3_ nanocomposite and anionic dye. Afterward, the electron is transferred by Ni/Cs@CMC/CuO-Co_2_O_3_ from BH_4_^−^ to EY for its catalytic reduction [4].

**Figure 11:**
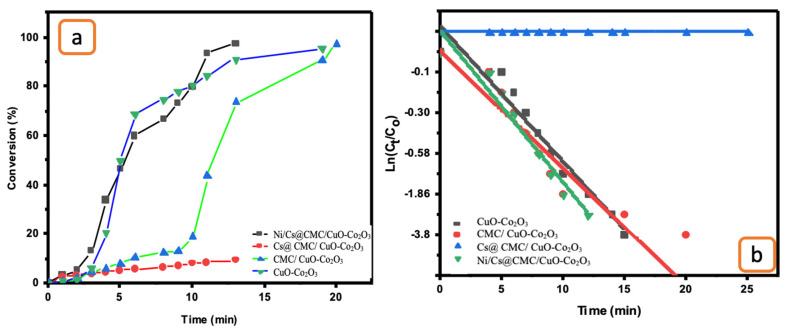
(**a**) Conversion versus time for the reduction of 4-NP by the prepared catalysts. (**b**) ln(C_t_/C_o_) vs. time for the transformation of 4-NP by the prepared catalysts.

Additionally, an evaluation of CuO-Co_2_O_3_, CMC/CuO-Co_2_O_3_, Cs@CMC/CuO-Co_2_O_3_ and Ni/Cs@CMC/CuO-Co_2_O_3_ as catalysts toward the catalytic reduction of 4-NP was performed. By using the same procedure mentioned previously, the reaction of 4-NP was performed by utilizing CuO-Co_2_O_3,_ CMC/CuO-Co_2_O_3_, Cs@CMC/CuO-Co_2_O_3_ and Ni/Cs@CMC/CuO-Co_2_O_3_ as catalysts in the presence of NaBH_4_. In the beginning, the absorbance band of 4-NP appeared at λ_max_ = 317 nm. As observed, the yellow color of 4-NP changed directly to dark yellow in the presence of (0.5 mL) NaBH_4_ with a new UV–Vis band appearing at 400 nm. This indicates the transformation of 4-NP to 4-nitrophenolate. Then, the CuO-Co_2_O_3,_ CMC/CuO-Co_2_O_3_, Cs@CMC/CuO-Co_2_O_3_ and Ni/Cs@CMC/CuO-Co_2_O_3_ catalysts were added and tested separately for the reduction of 4-nitrophenol. The band at λ_max_ = 400 nm disappeared gradually, and a new absorbance band at λ_max_ = 320 nm appeared along with the disappearance of dark yellow color, proving the formation of 4-AP because of 4-NP reduction. It was found that among all the catalysts, the prepared Ni/Cs@CMC/CuO-Co_2_O_3_ was the most effective catalyst because 4-NP was completely reduced to 4-AP in 13 min, while it was reduced in 19 and 20 min by using CuO-Co_2_O_3_ and CMC/CuO-Co_2_O_3_, respectively. However, the reduction of 4-NP by Cs@CMC/CuO-Co_2_O_3_ took 20 min (Figure 11a). 

The rate constant and R^2^ were found to be 4.26 × 10^−3^ s^−1^ and 0.912, 17 × 10^−5^ s^−1^ and 0.824, 2.62 × 10^−3^ s^−1^ and 0.842 and 2.7 × 10^−3^ s^−1^ and 0.855 for Ni/Cs@CMC/CuO-Co_2_O_3_, Cs@CMC/CuO-Co_2_O_3_, CMC/CuO-Co_2_O_3_ and CuO-Co_2_O_3_, respectively, as shown in Table 6 and Figure 11b. The data for 4-NP reduction was compared with other catalysts and is illustrated in Table 7.

**Figure 12:**
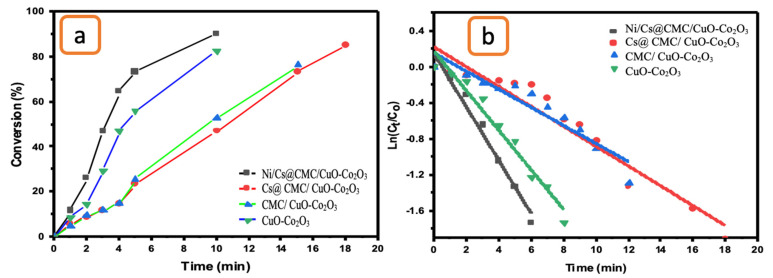
(**a**) Conversion versus time for the reduction of K_3_[Fe(CN)_6_] by the prepared catalysts. (**b**) ln(C_t_/C_o_) vs. time for the transformation of K_3_[Fe(CN)_6_] by the prepared catalysts.

The catalytic reduction of K_3_[Fe(CN)_6_] was also examined to evaluate the catalytic activity of CuO-Co_2_O_3_, CMC/CuO-Co_2_O_3_, Cs@CMC/CuO-Co_2_O_3_ and Ni/Cs@CMC/CuO-Co_2_O_3_. The UV-vis absorption of the catalytic reduction of K_3_[Fe(CN)_6_] was monitored every minute to check the progress of the K_3_[Fe(CN)_6_] reduction. As the catalytic reaction proceeded in the presence of NaBH_4_ and the catalyst, the absorption band of K_3_[Fe(CN)_6_] at λ_max_ = 420 nm gradually decreased in 6 min when using Ni/Cs@CMC/CuO-Co_2_O_3_ along with disappearance of the yellow color, indicating the reduction of K_3_[Fe(CN)_6_] to K_4_[Fe(CN)_6_] [69]. In contrast, the reduction reaction took longer times of 8, 13 and 18 min when using CuO-Co_2_O_3_, CMC/CuO-Co_2_O_3_ and Cs@CMC/CuO-Co_2_O_3,_ respectively. The efficient transformation of K_3_[Fe(CN)_6_] to K_4_[Fe(CN)_6_] was obtained by applying the Ni/Cs@CMC/CuO-Co_2_O_3_ catalyst (91%), while 85, 73.5 and 83% reduction were obtained by using Cs@CMC/CuO-Co_2_O_3_, CMC/CuO- Co_2_O_3_ and CuO-Co_2_O_3_, as shown in Figure 12a. Based on findings, the catalytic reduction reaction of K_3_[Fe(CN)_6_] follows the pseudo-first-order, as seen in Figure 12b. Subsequently, the rate constant and R^2^ were found to be 5.1 × 10^−3^ s^−1^ and 0.975, 1.8 × 10^−3^ s^−1^ and 0.909, 1.69 × 10^−3^ s^−1^ and 0.835 and 3.6 × 10^−3^ s^−1^ and 0.964 for Ni/Cs@CMC/CuO-Co_2_O_3_, Cs@CMC/CuO-Co_2_O_3_, CMC/CuO-Co_2_O_3_ and CuO-Co_2_O_3,_ respectively, as shown in Table 6.

The possible mechanism for the reaction of K_3_[Fe(CN)_6_] in the presence of both catalyst beads and NaBH_4_ is illustrated in Figure 10. Based on the reported studies, the catalytic reduction of [Fe(CN)_6_]^−3^ to form [Fe(CN)_6_]^−4^ is an electron-transfer route, as shown in the reaction below [3]. Therefore, the catalytic reaction mechanism of K_3_[Fe(CN)_6_] involves two main steps. Initially, polarization of the catalyst nanocomposite beads occurs by the reducing agent NaBH_4_. Afterward, e^−^ are transferred from the catalyst surface to the [Fe(CN)_6_]^−3^ and get reduced to [Fe(CN)_6_]^−4^, as shown in Equation (8):

BH_4_^−^ (aq) + 8[Fe(CN)_6_]^3−^ (aq) + 3H_2_O (aq) → H_2_BO_3_^−^ (aq) + 8[Fe(CN)_6_]^4−^ (aq) + 8H^+^ (8)

### 2.6. Catalytic Optimization

#### 2.6.1. Effect of Contaminant Concentrations

**Figure 13:**
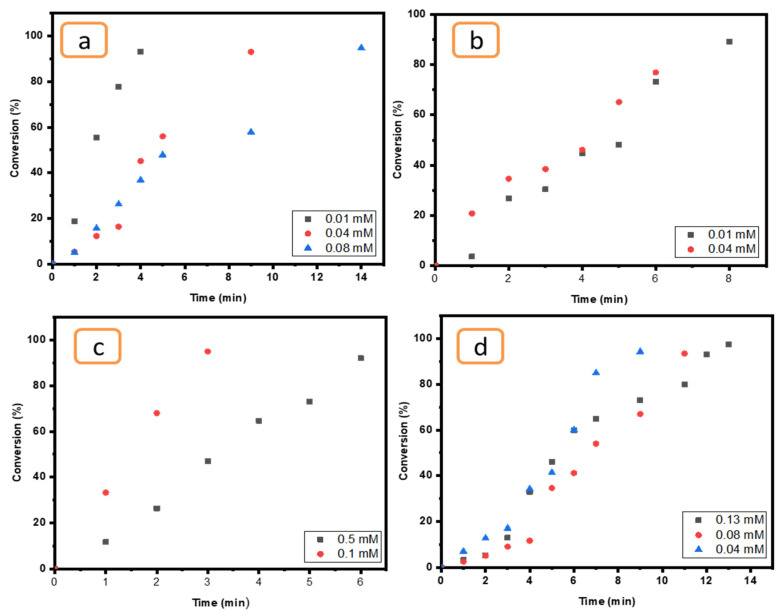
Effect of pollutants concentration for (**a**) MO (0.01, 0.04 and 0.08 mM), (**b**) EY (0.01 and 0.04 mM), (**c**) K_3_[Fe(CN)_6_] (0.1 and 0.5 mM) and (**d**) 4-NP (0.04, 0.08 and 0.1 mM) reduction using 5 mg of Ni/Cs@CMC/CuO-Co_2_O_3_ beads.

The effect of concentration was examined for all compounds (4-NP, EY, MO and K_3_[Fe(CN_6_)]) by using a certain catalyst (Ni/Cs@CMC/CuO-Co_2_O_3_) since it is more effective than others (Cs@CMC/CuO-Co_2_O_3,_ Cs@CMC/CuO-Co_2_O_3_, CMC/CuO-Co_2_O_3_ and CuO-Co_2_O_3_). As clearly seen in all figures below (Figure 13), when the concentration of pollutants was increased the time taken for reduction was increased. This finding indicates that the concentration of the pollutants has an essential role and Ni/Cs@CMC/CuO-Co_2_O_3_ was found to be more efficient catalyst with a low concentration of MO, EY, 4-NP and K_3_[Fe(CN)_6_], which was found to have similar effect as reported in literature [70].

#### 2.6.2. Effect of NaBH_4_ Concentration

**Figure 14:**
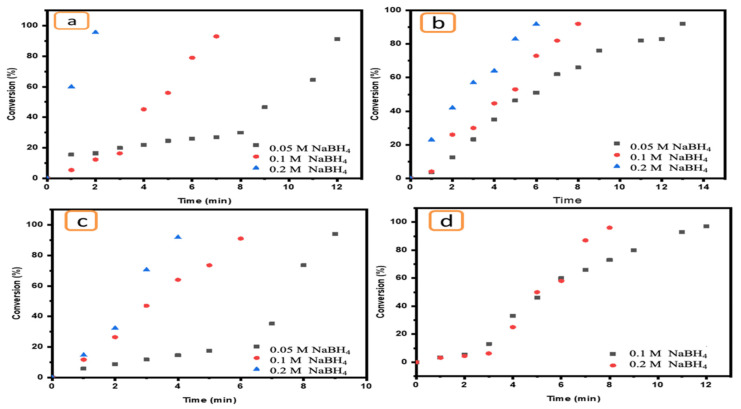
Effect of NaBH_4_ concentrations (0.2, 0.1 and 0.05 M) on the reduction of (**a**) MO, (**b**) EY, (**c**) K_3_[Fe(CN)_6_] and (**d**) 4-NP using 5 mg of Ni/Cs@CMC/CuO-Co_2_O_3_ beads.

The impact of NaBH_4_ concentration on the catalytic reduction of pollutants is a very important parameter. Therefore, a range of NaBH_4_ concentrations (0.2, 0.1 and 0.05 M) were used to evaluate its effect on the reduction of target pollutants in the presence of Ni/Cs@CMC/CuO-Co_2_O_3_ beads as a catalyst. As the data demonstrate, the reducing agent has an important role in the reduction reaction of pollutants in the presence of an effective catalyst. However, this reducing agent has no activity or ability to reduce toxic compounds even at high concentrations. Therefore, an effective catalyst should be added to enhance the reduction. A high concentration of NaBH_4_ (0.2 M) in addition to an effective catalyst such as Ni/Cs@CMC/CuO-Co_2_O_3_ can promote the reduction reaction of MO at a faster rate, decolorizing it in only 2 min, as shown in Figure 14a. However, when the reducing agent (NaBH_4_) concentration was decreased, the reduction reaction rate was influenced and decreased. This means that the reduction reaction requires more time to complete. Indeed, MO was reduced in 4 min and 12 min when the NaBH_4_ concentration was 0.1 and 0.05 M, respectively, as shown in Figure 14a. EY was also reduced in 6 min by using a high NaBH_4_ concentration, while this reduction took 9 min and 14 min using 0.1 and 0.05 M NaBH_4_, respectively, as shown in Figure 16b. The same effect was observed for K_3_[Fe(CN)_6_] (Figure 14c) and 4-NP (Figure 14d), and a similar impact was reported in literature [70]. 

#### 2.6.3. Effect of Number of Ni/Cs@CMC/CuO-Co_2_O_3_ Beads

**Figure 15:**
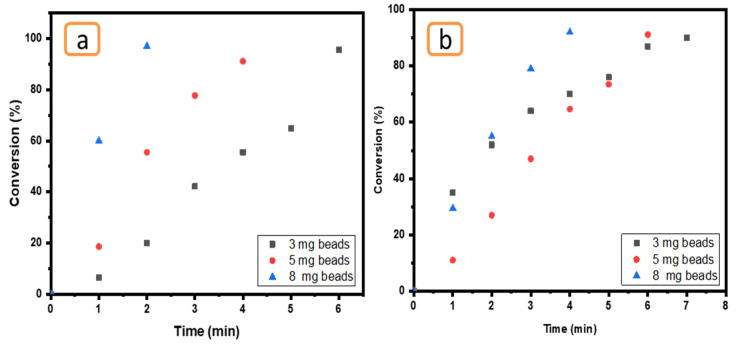
Effect of Ni/Cs@CMC/CuO-Co_2_O_3_ beads amount (3 mg, 5 mg and 8 mg) on the reduction of (**a**) MO and (**b**) K_3_[Fe(CN_6_)].

The influence of amount of Ni/Cs@CMC/CuO-Co_2_O_3_ was tested by utilizing three different amounts of Ni/Cs@CMC/CuO-Co_2_O_3_ bead catalyst (3 mg, 5 mg and 8 mg) in the presence of reducing agent (0.2 M NaBH_4_). This effect was tested using 0.01 mM MO and 0.05 mM K_3_[Fe(CN_6_)], as seen in Figure 15. In fact, the amount of catalyst is an important factor in the reduction reaction. The results demonstrate that a larger catalyst amount (8 mg of Ni/Cs@CMC/CuO-Co_2_O_3_) could enhance the reaction of MO causing decolorization by 98% in 2 min and 92% in 2 min using an amount of 5 mg. However, MO was reduced by 97% in 6 min by using a low amount of catalyst, as shown in Figure 15a. A similar effect was found for K_3_[Fe(CN_6_)], as clearly seen in Figure 15b.

### 2.7. Recyclability of Ni/Cs@CMC/CuO-Co_2_O_3_ Beads

**Figure 16:**
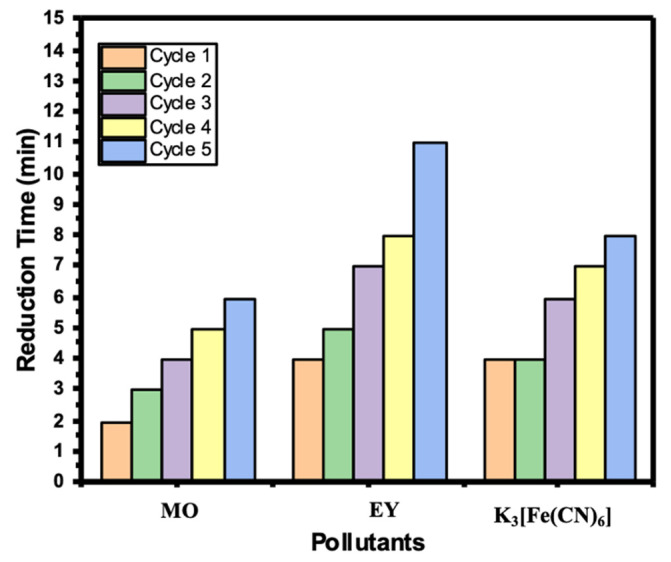
Recyclability of Ni/Cs@CMC/CuO-Co_2_O_3_ beads toward the reduction of MO, EY and K_3_[Fe(CN_6_)].

Recyclability of the catalyst is a significant factor during a catalytic reduction study. Most of the catalysts become deactivated after the first or second use. In our study, Ni/Cs@CMC/CuO-Co_2_O_3_ was able to be reused up to five times without deactivation or any loss of the catalyst beads. Consequently, Ni/Cs@CMC/CuO-Co_2_O_3_ beads were tested in the reduction of MO, EY and K_3_[Fe(CN)_6_] for several time to check the recyclability of the catalyst. As mentioned previously, the same procedures were followed, except the beads were washed by deionized water and then MeOH, followed by deionized water several times, and then dried for the next use. This process was repeated five times to assess the reusability of the Ni/Cs@CMC/CuO-Co_2_O_3_ beads. Figure 16 shows the time taken for each reduction cycle of MO, 4-NP and K_3_[Fe(CN)_6_] using Ni/Cs@CMC/CuO-Co_2_O_3_ beads.

### 2.8. Application to Real Samples

The catalytic activity of Ni/Cs@CMC/CuO-Co_2_O_3_ beads was also assessed in four types of real samples with spiked MO (0.06 mM). The real samples used for this study were full-fat milk and three juice samples (orange, pineapple and apple), and they were obtained from a local market (Jeddah, Saudi Arabia). The preparation of real samples was performed by taking around 1 mL of each sample and diluting it in 100 mL of deionized water individually. Further, 2.5 mL of each real sample was placed into a UV-vis cuvette, and 0.5 mL of 0.06 mM MO was added followed by addition of 0.5 mL of 0.1 M NaBH_4_. Finally, 5 mg of Ni/Cs@CMC/CuO-Co_2_O_3_ was added. The catalytic degradation of MO was monitored by a UV-vis spectrophotometer. As clearly seen from data presented in Table 8, full-fat milk was the only sample that took a longer time (15 min) with a very low reduction % (65%). This is due to the high interference found in the milk, which can influence the reduction of MO. In contrast, the reduction of MO in the three juice samples occurred in 5–6 min with 91–97%. The data confirm that the Ni/Cs@CMC/CuO-Co_2_O_3_ is effective and reliable since it was able to decolorize and effectively reduce MO in real samples.

## 3. Conclusions

CuO-Co_2_O_3_ was synthesized, mixed with CMC and turned into beads by the crosslinking agent (AlCl_3_) before being coated with chitosan. XRD, FT-IR, SEM and EDX were used to characterize CuO-Co_2_O_3_, CMC/CuO-Co_2_O_3_, Cs@CMC/CuO-Co_2_O_3_ and CuO-Co_2_O_3_. The findings confirm that Cs@CMC/CuO-Co_2_O_3_ is an efficient adsorbent toward Ni ions compared to other selected metal ions. Additionally, Ni/Cs@CMC/CuO-Co_2_O_3_ was used as a catalyst for degradation of MO and EY as well as catalytic reduction of K_3_[Fe(CN)_6_] and 4-NP. This catalyst could be recycled and was able to be used up to five times, confirming the effectiveness and the stability of catalyst.

## 4. Experimental

### 4.1. Materials

Metal salts of CoSO_4_·6H_2_O (99.998%), CuSO_4_·5H_2_O (≥98.0%), Ni(NO_3_·6H_2_O, (99.999%) FeSO_4_·7H_2_O (≥99.0%), AgNO_3,_ ZnSO_4_·7H_2_O (≥99.0%) and AlCl_3_ (99.999%) were obtained from Sigma-Aldrich. Biopolymers: chitosan and carboxymethylcellulose were obtained from BDH chemicals. Organic compounds including dyes (Methyl Orange (MO, (85 %)) and Eosin yellowish (EY, (5 wt.% in H_2_O)) and 4-nitrophenol (4-NP, (≥99%)) were obtained from Sigma-Aldrich. Potassium hexacyanoferrate (III), (99%) and the reducing agent (sodium borohydride, (99%)) were obtained from Sigma-Aldrich. In all preparations, deionized water was utilized.

### 4.2. Synthesis of Nanocomposites and Beads

#### 4.2.1. Preparation of CuO-Co_2_O_3_ Nanocomposite

The nanocomposite, CuO-Co_2_O_3_ was prepared by co-precipitation method. Firstly, 0.1M CuSO_4_·5H_2_O was mixed with 0.1 M CoSO_4_·6 H_2_O in a (50:50) ratio. Then, NaOH was added dropwise to adjust the pH, 10–11. The preparation was carried out at 80 °C with stirring for 4 h. Finally, the precipitate was collected via filtration, then washed several times with deionized water and dried overnight. Afterward, the CuO-Co_2_O_3_ nanocomposite was calcined at 500 °C for 5 h.

#### 4.2.2. Synthesis of Cs@CMC/CuO-Co_2_O_3_ Beads

**Figure 17:**
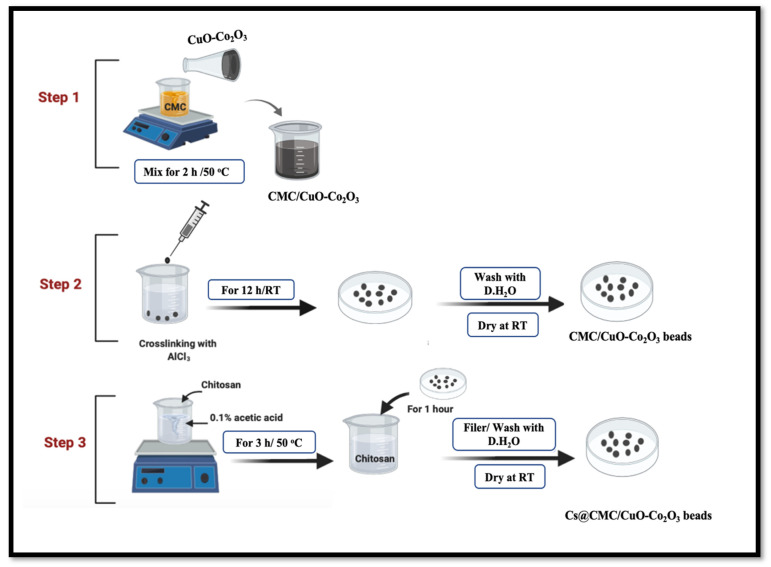
Preparation of CMC/CuO-Co_2_O_3_ and Cs@CMC/CuO-Co_2_O_3_ nanocomposite beads. CMC was dissolved in (25 mL) with stirring for 2 h under 50 °C. CuO-Co_2_O_3_ was dissolved in 5 mL of D.H_2_O and sonicated for around 10 min. CuO-Co_2_O_3_ solution was mixed with CMC solution at 50 °C.

The novel nanocomposite beads were synthesized in two main steps. The method was reported by our group with some modifications [4,21]. The first step, CMC (0.5 g) was dissolved in (25 mL) with stirring for 2 h at 50 °C. Meanwhile, 60 mg of CuO-Co_2_O_3_ powder was dissolved in 5 mL of deionized water and further sonicated for around 10 min for suspension of CuO-Co_2_O_3_. Subsequently, CuO-Co_2_O_3_ dispersed solution was added into CMC solution with continuous stirring for 1 h at 50 °C, and half an hour at RT (24 °C). The mixture was transferred to 3 mL syringe and dropped into 0.2 M AlCl_3_ solution for crosslinking and formation of beads. The beads were kept in AlCl_3_ solution for 12 h and then collected and washed three times using deionized water. Secondly, Cs solution was prepared in 1% acetic acid distilled water mixture and stirred for 3 h at 50 °C. The washed beads were transferred to Cs solution and kept for 1 h. Finally, the Cs@CMC/CuO-Co_2_O_3_ beads were separated and dried at RT for 24 h. Dry CMC/CuO-Co_2_O_3_ beads were flat, while the dry Cs@CMC/CuO-Co_2_O_3_ beads had a circle shape due to their chitosan coating, Figure 17. 

### 4.3. Metal Uptake Adsorption

In order to evaluate the selectivity of the prepared nanocomposite beads (Cs@CMC/CuO-Co_2_O_3_ and CMC/CuO-Co_2_O_3_), adsorption methods of metal ions were developed toward certain metal ions including Ni(II), Ag(I), Zn(II), and Fe(II). Certain amounts of Cs@CMC/CuO-Co_2_O_3_ and CMC/CuO-Co_2_O_3_ (5.0 mg) were added individually into 5 mL of 5 ppm of sample solution of selected metal ions for 1 h at RT (25 ± 1 °C). The beads were separated from the solutions and dried at RT. The inductively coupled plasma−optical emission spectroscopy (ICP-OES) was employed to detect the concentration of each metal ion before and after adsorption on Cs@CMC/CuO-Co_2_O_3_ and CMC/CuO-Co_2_O_3_ beads. Optimization of parameters of the selected metal ions Ni(II) was carried out as mentioned later. Moreover, the Ag (I) beads were also kept after adsorption and converted to NPs for further studies.

For isotherm study, 5 mg of each adsorbent Cs@CMC/CuO-Co_2_O_3_ and CMC/CuO-Co_2_O_3_ beads was added to 5 mL of Ni solution with initial concentrations from (5–100 mgL^−1^). The pH value of Ni solution was adjusted to pH = 7, with mechanical shaking for 60 min.

Moreover, for kinetic study, 5 mg of each adsorbent Cs@CMC/CuO-Co_2_O_3_ and CMC/CuO-Co_2_O_3_ bead was added to 5 mL of 5 mgL^−1^ Ni solution. The pH value of Ni solution was adjusted to pH = 7. The concentration of Ni ions was tested at different times (10, 30, 60, 120, 240 min).

### 4.4. Formation of Zero-Valent Nanoparticles

**Figure 18:**
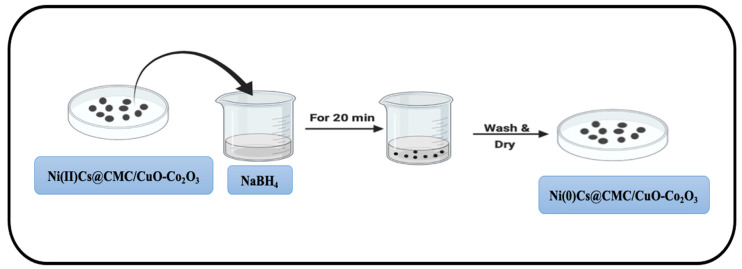
Reduction of Ni(II)Cs@CMC/CuO-Co_2_O_3_ beads to Ni(0)Cs@CMC/CuO-Co_2_O_3_.

Ni(II)/Cs@CMC/CuO-Co_2_O_3_ and Ag(I)/CMC/CuO-Co_2_O_3_ beads were collected and dried. These dried beads were later utilized for the synthesis of nanoparticles (Figure 18) by loading them into a freshly prepared 0.05 M NaBH_4_ aqueous solution for 20 min in order to complete the reduction of Ni(II) and Ag(I) to Ni(0) and Ag(0) zero-valent nanoparticles, respectively, as shown in the following Equations (9) and (10).

M^2+^ + 4BH_4_^−^ + 12H_2_O → 2M^0^ + 14H_2_ + 4B(OH)_3_ (9)

M^+^ + 2BH_4_^−^ + 6H_2_O → M^0^ + 7H_2_ + 2B(OH)_3_ (10)

### 4.5. Catalytic Reduction Experiments

The catalytic ability of prepared catalysts, Ni/Cs@CMC/CuO-Co_2_O_3_, Cs@CMC/CuO-Co_2_O_3_, CMC/CuO-Co_2_O_3_ and CuO-Co_2_O_3_, was investigated toward organic and inorganic pollutants. Firstly, 0.01 mM MO and EY, 0.1 mM 4-NP and 0.5 mM K_3_[Fe(CN)_6_] were tested using 0.1 M NaBH_4_ and 5 mg of each prepared catalyst individually, including Ni/Cs@CMC/CuO-Co_2_O_3_,Cs@CMC/CuO-Co_2_O_3_, CMC/CuO-Co_2_O_3_ and CuO-Co_2_O_3_. Different concentrations of MO and EY (0.01, 0.04 and 0.08 mM), 4-NP (0.1 and 0.04, 0.08 mM) and K_3_[Fe(CN)_6_] (0.1 mM, 0.5 mM) were then examined by utilizing Ni/Cs@CMC/CuO-Co_2_O_3_ nanoparticles beads. Additionally, different concentrations of NaBH_4_ (0.05, 0.1 and 0.2 M) were tested using 0.01 mM MO and EY, 0.1 mM 4-NP and 0.5 mM K_3_[Fe(CN)_6_] with 5 mg of Ni/Cs@CMC/CuO-Co_2_O_3_ nanoparticles bead catalyst. Moreover, different amounts (3, 5 and 8 mg) of Ni/Cs@CMC/CuO-Co_2_O_3_ beads were tested using 0.01 mM MO and 0.5 mM K_3_[Fe(CN)_6_] with 0.2 M NaBH_4_.

The catalytic procedure was performed by placing 2.5 mL of each pollutant (MO, EY, 4-NP and K_3_[Fe (CN)_6_]) in a UV cuvette, and 0.5 mL of a freshly prepared solution of NaBH_4_ (0.1 M) was added. Then, 5 mg of the prepared catalyst and NaBH_4_ were both added to the mixture in the cuvette. Catalytic activity was continuously examined via UV-vis spectrophotometer with every 1 min interval. The recyclability of Ni/Cs@CMC/CuO-Co_2_O_3_ beads was tested in the catalytic reduction of MO, EY and K_3_[Fe(CN)_6_]_._ This catalyst was used up to five times after washing with deionized water and MeOH and dried for the next cycle.

The conversion (%) of all compounds was calculated by utilizing Equation (11):

(11) $$\%R=\frac{C_{0}-C_{t}}{C_{0}}*100$$

where *C_0_* (mgL^−1^) is the initial concentration of compounds, and *C_e_* (mgL^−1^) is the final concentration.

The rate constant *K* and adjacent *R^2^* values were determined from pseudo-first-order kinetics as described below in Equation (12):

(12) $$\ln\frac{C_{t}}{C_{0}}=-Kt$$

### 4.6. Characterization

The morphologies and structures of Ni/Cs@CMC/CuO-Co_2_O_3_, Cs@CMC/CuO-Co_2_O_3_, CMC/CuO-Co_2_O_3_ and CuO-Co_2_O_3_ were characterized by scanning electron microscope (SEM) (of JEOL, JSM-7600F, Japan). For SEM analysis, Ni/Cs@CMC/CuO-Co_2_O_3_, Cs@CMC/CuO-Co_2_O_3_, CMC/CuO-Co_2_O_3_ and CuO-Co_2_O_3_ were individually fixed on the stub using carbon tape as a binder and then sputtered with platinum for 15 s. In addition, X-ray diffraction (XRD) was employed to examine the phase structure of all prepared catalysts. Elemental analysis of Ni/Cs@CMC/CuO-Co_2_O_3_, Cs@CMC/CuO-Co_2_O_3_, CMC/CuO-Co_2_O_3_ and CuO-Co_2_O_3_ was analyzed by energy dispersive spectrometer (EDS). FTIR spectrometer was applied to analyze the spectra of all prepared materials. The catalytic reduction studies were recorded by UV–vis spectra, (Thermo Scientific TM Evolution TM 350 UV–vis spectrophotometer).

## Figures and Tables

**Table 1:** Uptake capacity of Ni, Fe, Zn and Ag ions on CMC/CuO-Co_2_O_3_ and Cs@ CMC/CuO-Co_2_O_3_ beads.

Metal Ions	Wavelength Detection (nm)	Initial Conc. (mgL^−1^)	CMC/CuO-Co_2_O_3_				Cs@CMC/CuO-Co_2_O_3_			
			Final Conc. (mgL^−1^)	q_e_ (mgg ^−1^)	K_d_ (mLg^−1^)	R (%)	Final Conc. (mgL^−1^)	q_e_ (mgg^−1^)	K_d_ (mLg^−1^)	R (%)
Ni(II)	221.648	5	4.8	0.2	41.666	4	0.697	4.3	6173.6	86.06
Zn(II)	213.9	5	4.6	0.4	86.956	8	1.5	3.5	2333.3	70
Fe(II)	248	5	4.4	0.6	136.363	12	2	3	1500	60
Ag(I)	243.778	5	0.23	5	20,739.1	95.4	1.7	3.3	1941.1	66

**Table 2:** Mathematical equations and isotherm models [49,50,51] used in this study.

	Models	Linear Equations	Plot
Isotherm	Langmuir	$\frac{C_{e}}{q_{t}}=\frac{1}{q_{mK_{L}}}+\frac{C_{e}}{q_{m}}$(4)	$\frac{C_{e}}{q_{t}}\;vs.\;C_{e}$
	Freundlich	$logq_{e}=\log K_{f}+\frac{1}{n}\log C_{e}$ (5)	$logq_{e}vs.\log C_{e}$
Kinetic	Pseduo-first-order	$log\left(q_{e}-q_{t}\right)={\;\mathrm{logq}}_{e}-\left(k_{1}/2.303\right)t$ (6)	$log\left(q_{e}-q_{t}\right)\;vs.\;t$
	Pseduo-second-order	$\frac{t}{q_{t}}=\frac{1}{v_{o}}+\frac{1}{q_{e}}t$ (7)	$\frac{t}{q_{t}}\;vs.\;t$

**Table 3:** Data of isotherm models for Ni(II) adsorption using Cs@CMC/CuO-Co_2_O_3_.

	Langmuir Model				Freundlich Model		
Metal Ion	$q_{max}$	R^2^	$R_{L}$	b (Lmg^−1^)	R^2^	$k_{f}$	n
Ni(II)	12.00	0.943	0.50	0.08	0.553	9.10	5.06

**Table 4:** Kinetic models data for Ni(II) adsorption by using 5 mg of Cs@CMC/CuO-Co_2_O_3_.

	$q_{e}$ (mg g^−1^) Experiment	Pseudo-First-Order			Pseudo-Second-Order		
Metal Ion		R^2^	K_1_ (min^−1^)	$q_{e}$ mg g^−1^	R^2^	K_2_ (g mg^−1^min^−1^)	$q_{e}$ mg g^−1^
Ni(II)	11.00	0.703	0.0011	2.72	0.986	−0.222	4.05

**Table 5:** Comparison of this study with other reported studies in literature.

Adsorbent	Metal Ion	Condition	q_max_ (mgg^−1^)	Ref.
CS/TEOS/APTES	Ni(II)	pH 5.2, 30 °C, 30 min	696.2	[48]
Chitosan-g-maleic acid	Ni(II)	pH 8, room temperature, 60 min	73.5	[26]
Flower globular FGMH	Ni(II)	pH 6–7, 20 °C, 50 min	248	[52]
Chitosan-MOF	Ni(II)	pH 5, 20 °C, 480 min	56	[53]
Cs@CMC/CuO-Co_2_O_3_	Ni(II)	pH 7, room temperature, 60 min	11	This study

**Table 6:** Rate constant and R^2^ for the degradation of EY and MO and reduction of 4-NP and K_3_[Fe(CN)_6_].

	Rate Constant K (s^−1^) and Adjacent R^2^ Value			
Compound	CuO-Co_2_O_3_	CMC/CuO-Co_2_O_3_	Cs@CMC/CuO-Co_2_O_3_	Ni/Cs@CMC/CuO-Co_2_O_3_
MO	6.11 × 10^−3^ and 0.865	2.6 × 10^−3^ and 0.915	4.4 × 10^−4^ and 0.953	1.06 × 10^−2^ and 0.964
EY	2.95 × 10^−4^ and 0.989	3.2 × 10^−3^ and 0.928	7.71 × 10^−4^ and 0.968	4.58 × 10^−3^ and 0.936
4-NP	2.7 × 10^−3^ and 0.855	2.62 × 10^−3^ and 0.842	1.7 × 10^−5^ and 0.824	4.26 × 10^−3^ and 0.912
K_3_[Fe(CN)_6_]	3.6 × 10^−3^ and 0.964	1.69 × 10^−3^ and 0.835	1.8 × 10^−3^ and 0.909	5.1 × 10^−3^ and 0.975

**Table 7:** Comparison of Ni/Cs@CMC/CuO-Co_2_O_3_ catalyst toward the reduction of MO, EY, 4-NP and K_3_[Fe(CN)_6_] with other reported catalysts.

Pollutant	Catalyst	Time (min)	Rate Constant	Ref.
MO	Ni/Cs@CMC/CuO-Co_2_O_3_	2	1.06 × 10^−2^	This study
MO	TO-CoNPs	60	-	[60]
MO	AuNPs	8	1.7 × 10^−3^	[61]
MO	WBs loaded with Ni NPs	15	2.37 × 10^−3^	[62]
EY	Ni/Cs@CMC/CuO-Co_2_O_3_	6	4.58 × 10^−3^	This study
EY	Chitosan-capped AuNPs	50	-	[63]
4-NP	Ni/Cs@CMC/CuO-Co_2_O_3_	13	4.26 × 10^−3^	This study
4-NP	CMC-Cu	35	9.1 × 10^−4^	[64]
4-NP	PdNPs doped chitosan	15	3.63 × 10^−3^	[65]
4-NP	Ni/CS-FP	28	-	[66]
K_3_[Fe(CN)_6_]	Ni/Cs@CMC/CuO-Co_2_O_3_	6	5.1 × 10^−3^	This study
K_3_[Fe(CN)_6_]	NiWO_4_ Nanoparticles	240	-	[67]
K_3_[Fe(CN)_6_]	Fe_3_O_4_-CuAg NPs	3	19.3 × 10^−3^	[68]
K_3_[Fe(CN)_6_]	CMC/CuO-NiO	0.5	6.88 × 10^−2^	[4]

**Table 8:** Application of four real samples spiked with MO.

Real Sample	Reduction Time (min)	Reduction %
Full-Fat Milk	15 min	65.5
Orange Juice	6 min	91.4
Pineapple Juice	5 min	95
Apple Juice	5 min	97

## Data Availability

The data presented in this study are available on request from the corresponding author.
